# Supplementary material for: Splice-Junction-Based Mapping of Alternative Isoforms in the Human Proteome
Source: Cell Rep. Author manuscript; Available in PMC 2020 Jan 15. (PMC6961840; doi:10.1016/j.celrep.2019.11.026)
Supplement: 3 [file NIHMS1546469-supplement-3.zip › DF2/PXD009737/TestisGluC-251-Q12923-SDSSSIEDPGQAYVLD.pdf]

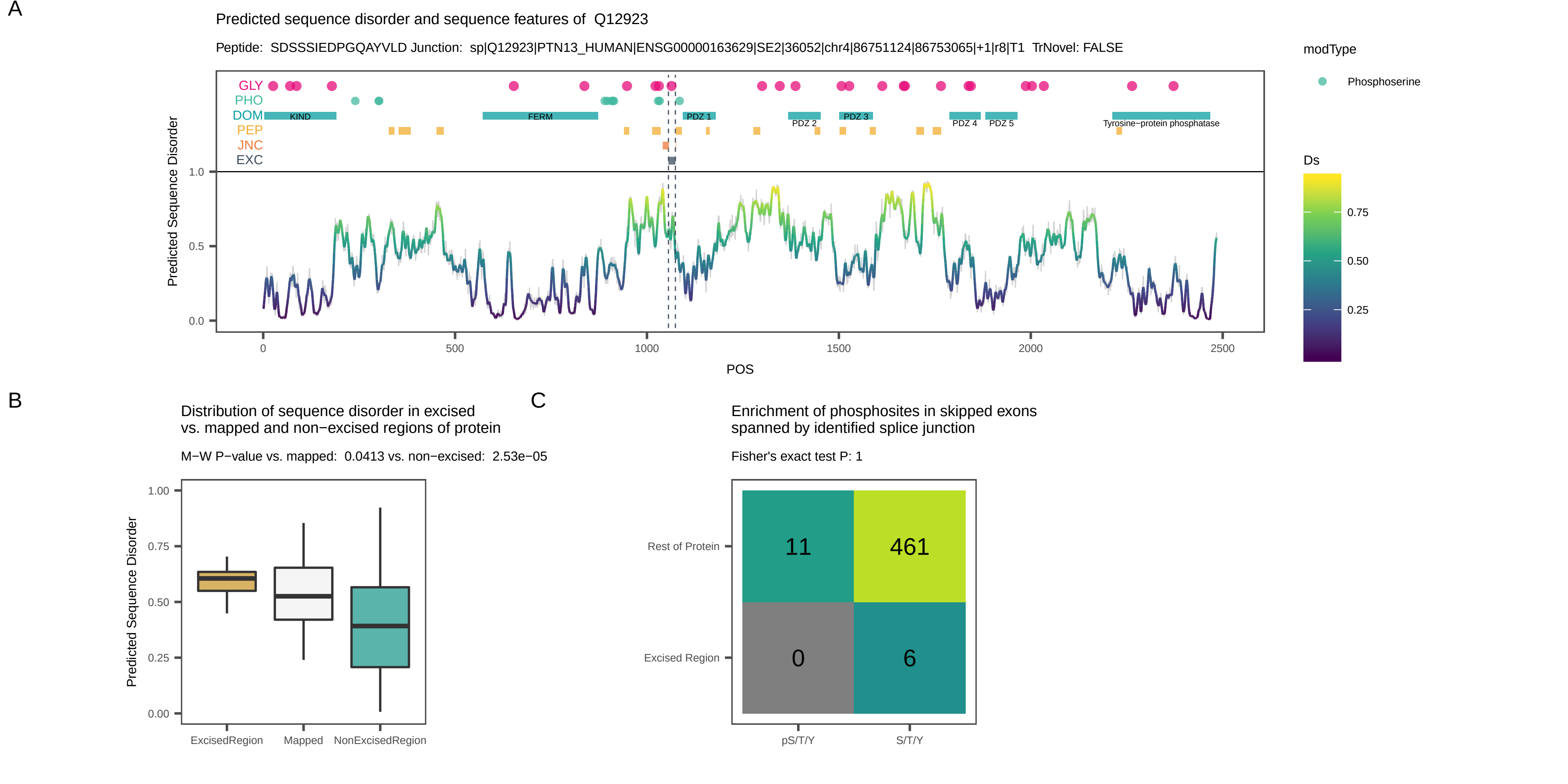
A
Predicted sequence disorder and sequence features of Q12923
Peptide: SDSSSIEDPGQAYVLD Junction: sp|Q12923|PTN13_HUMAN|ENSG00000163629|SE2|36052|chr4|86751124|86753065|+1|r8|T1 TrNovel: FALSE
GLY
PHO
DOM
PEP
JNC
EXC
KIND
FERM
PDZ 1
PDZ 2
PDZ 3
PDZ 4
PDZ 5
Tyrosine−protein phosphatase
Predicted Sequence Disorder
1.0
0.5
0.0
0
500
1000
1500
2000
2500
POS
modType
Phosphoserine
Ds
0.75
0.50
0.25
B
Distribution of sequence disorder in excised
vs. mapped and non−excised regions of protein
M−W P−value vs. mapped: 0.0413 vs. non−excised: 2.53e−05
Predicted Sequence Disorder
1.00
0.75
0.50
0.25
0.00
ExcisedRegion
Mapped
NonExcisedRegion
C
Enrichment of phosphosites in skipped exons
spanned by identified splice junction
Fisher's exact test P: 1
Rest of Protein
Excised Region
11
461
0
6
pS/T/Y
S/T/Y
